# The assessment of efficient representation of drug features using deep learning for drug repositioning

**DOI:** 10.1186/s12859-019-3165-y

**Published:** 2019-11-14

**Authors:** Mahroo Moridi, Marzieh Ghadirinia, Ali Sharifi-Zarchi, Fatemeh Zare-Mirakabad

**Affiliations:** 1 Department of Mathematics and Computer Science, Amirkabir University of Technology, (Tehran Polytechnic), Tehran, Iran; 2 Department of Computer Engineering, Sharif University of Technology, Tehran, Iran

**Keywords:** Drug indication prediction, Drug repurposing, Deep neural network

## Abstract

**Background:**

De novo drug discovery is a time-consuming and expensive process. Nowadays, drug repositioning is utilized as a common strategy to discover a new drug indication for existing drugs. This strategy is mostly used in cases with a limited number of candidate pairs of drugs and diseases. In other words, they are not scalable to a large number of drugs and diseases. Most of the in-silico methods mainly focus on linear approaches while non-linear models are still scarce for new indication predictions. Therefore, applying non-linear computational approaches can offer an opportunity to predict possible drug repositioning candidates.

**Results:**

In this study, we present a non-linear method for drug repositioning. We extract four drug features and two disease features to find the semantic relations between drugs and diseases. We utilize deep learning to extract an efficient representation for each feature. These representations reduce the dimension and heterogeneity of biological data. Then, we assess the performance of different combinations of drug features to introduce a pipeline for drug repositioning. In the available database, there are different numbers of known drug-disease associations corresponding to each combination of drug features. Our assessment shows that as the numbers of drug features increase, the numbers of available drugs decrease. Thus, the proposed method with large numbers of drug features is as accurate as small numbers.

**Conclusion:**

Our pipeline predicts new indications for existing drugs systematically, in a more cost-effective way and shorter timeline. We assess the pipeline to discover the potential drug-disease associations based on cross-validation experiments and some clinical trial studies.

## Background

De novo drug discovery procedure is time-consuming and expensive. More than 90% of drugs fail during the development stages due to inefficacy or high toxicity [1, 2]. To overcome these challenges, researchers are interested in finding a method to discover new drug-disease associations based on known drugs. The process of identifying new indications for existing drugs is known as drug repositioning (repurposing) [3–5]. In the last decade, several efforts have been made to find an efficient computational solution for drug repositioning [2, 6].

In biological experimental drug repositioning methods, it is hard to find new drug indications based on a large number of existing drugs due to low knowledge of biological mechanisms [7]. These methods are utilizable in most cases with the limited number of existing drugs and diseases pairs. In other words, they are not scalable to a large number of drugs and diseases. While computational approaches use the high-level integration of available drug and disease data to discover new drugs for human diseases [8]. By optimizing these strategies into efficient drug repositioning pipeline, repurposed drugs can be found systematically, in a much more cost-effective way and shorter timeline.

According to [2, 7, 9] there are five common categories for computational drug repositioning approaches named signature-based, network-based, text mining, semantic and machine learning algorithms.

One signature-based approach called ‘signature reversion’ [10, 11] looks for inverse drug-disease relationships by comparing disease-gene expression profiles and drug-gene expression profiles using CMAP [12], LINCS [13], and GEO [14] datasets. Another approach is defined based on ‘guilt-by-association’ principle which is applied to identify new targets for already approved drugs using DvD [15], DAVID [16] and GSEA repositories [10, 17, 18].

Zhang et al. [19] proposed a network-based approach using a unified framework for integrating multiple aspects of drug similarity and disease similarity. In this regards, they integrated genome (e.g., drug target protein, disease gene), phenome (e.g., disease phenotype, drug side effect), and drug chemical structure to extract the drug similarity network and the disease similarity network. Finally, a drug-disease network was constructed to explore novel drug indications. Yang et al. [20] utilized a causal inference-probabilistic matrix factorization approach to infer drug-disease associations. They integrated systematic multilevel relations to construct causal networks connecting drug–target–pathway–gene–disease. Lee et al. [21] constructed a directed network using protein interaction and gene regulation data obtained from various public databases providing diverse biological pathways for obtaining associations between drug and disease genes. They have employed interaction on the binary protein-protein interaction network with consideration to the characteristics of the interactions.

Extracting novel and valuable biological entity relations from the literature is challenging. Text mining techniques are widely used to solve it and identify connections between biological concepts or biological entities [22].

Semantic-based approach has been applied to drug repositioning in three main steps as follows: extracting and integrating public resources, constructing a semantic network by integrating multisource data and mining semantic links [7]. Mullen et al. [23] used a Bayesian statistics approach to rank drug-disease relationships according to prior knowledge. Then, they integrated ranked relationships with other biological entity associations to construct a semantical drug discovery network. To infer drug-disease relationships, the author applied an algorithm for detecting semantic subgraphs. Furthermore, Zhu et al. [24] proposed an automatic reasoning approach for heterogeneous semantics networks. Biological entities (such as drugs) are converted to labels in a semantic network. Then, disease-drug relationships are obtained from automatic reasoning techniques.

Predicting novel associations between drugs and diseases using the assorted data resources manually may not be efficient. Therefore, several machine learning methods have been proposed to solve this problem by extracting various features. Napolitano et al. [25] used drug-related features including drug chemical structure, drug molecular targets, and drug-induced gene expression signatures. These features were used to compute drug dissimilarity matrices merged into a single dissimilarity matrix as a kernel for support vector machine classification. Wang et al. [26] introduced an integrated model named ‘PreDR’ that trained an SVM model by incorporating drug chemical structure, side effect, and molecular activity.

In the first large-scale prediction of drug indications, Gottlieb et al. [5] utilized various disease-related and drug-related features. They constructed disease-disease similarity matrices by computing disease-disease similarity measures based on disease-related features called genetic and phenotypic signatures [27]. Furthermore, they studied many drug-related features like chemical structure, side effects, drug targets (sequence based), protein-protein interaction, and gene ontology [28]. Then, drug-drug similarity matrices were computed by the drug-drug similarity measures for each feature. Afterward, they trained a logistic regression classifier using known drug-disease associations. Finally, this classifier was used for new drug-disease association prediction [29]. Furthermore, Zhang et al. [30] proposed a similarity constrained matrix factorization method based on the biological context of the drug-disease association prediction (SCMFDD). In order to uncover latent features for drugs and diseases, SCMFDD projects the drug-disease associations into two low-rank spaces. Moreover, drug feature-based similarity and disease semantic similarity were introduced as constraints for drugs and diseases in the low-rank spaces. Xuan et al. [31] introduced a non-negative matrix factorization model called DisDrugPred for integrating drug similarity and disease similarity to predict drug–disease associations.

Most of the in-silico methods such as SCMFDD [30] and PREDICT [5] mainly focus on linear approaches while non-linear approaches are still scarce for new indication predictions [32]. Therefore, applying non-linear computational approaches can offer an opportunity to predict the possible drug repositioning candidates. For example, Donner et al. [33] trained a large data set of cellular perturbations using deep embedding of gene expression profiles. In addition, Zhao et al. [4] applied various state-of-the-art machine learning approaches for prediction, including deep neural networks, support vector machines, elastic net, random forest and gradient boosted machines for schizophrenia, depression and anxiety disorders.

Furthermore, the amount of biomedical data in freely available repositories is swiftly increasing. The nature of this data is heterogeneous, high-dimensional and noisy [34]. Consequently, designing an effective non-linear method like neural network for analyzing this data becomes more and more difficult [35, 36]. As a result, there is an urgent need for a more efficient representation of this data for integrative analysis. According to the key role of data representation, there is a large volume of studies describing the role of efficient representations for biological data [37]. We use some of these efficient representations derived by non-linear methods in order to reduce the dimension and heterogeneity of our biological features for the downstream analysis.

In this study, we present a pipeline to assess efficient representations of drug and disease features for drug indication prediction. In this regards, we introduce two similarity matrices to show the similarity between drug-drug and disease-disease pairs. Afterward, we train a classifier based on the similarity matrices to score each drug-disease pair. To construct the similarity matrices for the drug-drug and disease-disease pairs, we extract some biological features including chemical structures, protein sequences of drug target, drug-related enzyme sequences, and gene expression profiles for drugs, and also genotype and phenotype for diseases. To find an appropriate and continuous representation for chemical structures and sequences of proteins and enzymes, we utilize deep neural networks designed by Gómez-Bombarelli et al. [38] and Asgari et al. [39], respectively. Also, we design an auto-encoder to reduce the dimensionality of the gene expression profiles for better representation. We use principal component analysis (PCA) to reduce the dimensions of disease features (phenotype and genotype) represented by one-hot-encoder.

This paper demonstrates that the appropriate representation derived by deep learning leads to reasonable performance in drug repurposing. To assess the efficiency of feature representation, we employ and compare each subset of drug features (SDF) for drug repositioning. To make the drug-drug similarity matrix for each SDF, we extract a list of drugs from database where all features in the SDF are available. In other words, a small size of SDF leads to the selection of a large number of drugs and vice versa. These matrices are named drug-drug similarity intersection (DDSI) matrices. The results show that each SDF can find semantic relations between drugs and disease. Therefore, the proposed method is dependent on drug features representation and the number of drugs. Also, we construct the disease-disease similarity (DiDiS) matrix based on phenotype and genotype. Finally, drug-disease association (DDA) matrices are constructed based on DDSI, DiDiS matrices and known drug-disease associations set which are already clinically approved by regulatory agencies such as the US Food and Drug Administration.

A cross-validation scheme is used to find the best subset of drug features for drug repositioning. Our method achieves an area under the ROC curve 0.944. In addition, we assess each subset of drug features to find out: which drugs are effective for a specific disease and which diseases are treatable by a particular drug. Meanwhile, we compare our pipeline to Yang & Agarwa1 [40] and Lee [21] models on some specific diseases. In the following, we apply five-fold cross-validation to compare our method to PREDICT [5], SCMFDD [30] and DisDrugPred [31]. Finally, we suggest some new drug indications. We believe that our study is a step toward understanding the effect of drug feature representation on drug repositioning and inferring how each subset of drug features influences on drug indication for a specific disease.

## Methods

**Fig. 1:**
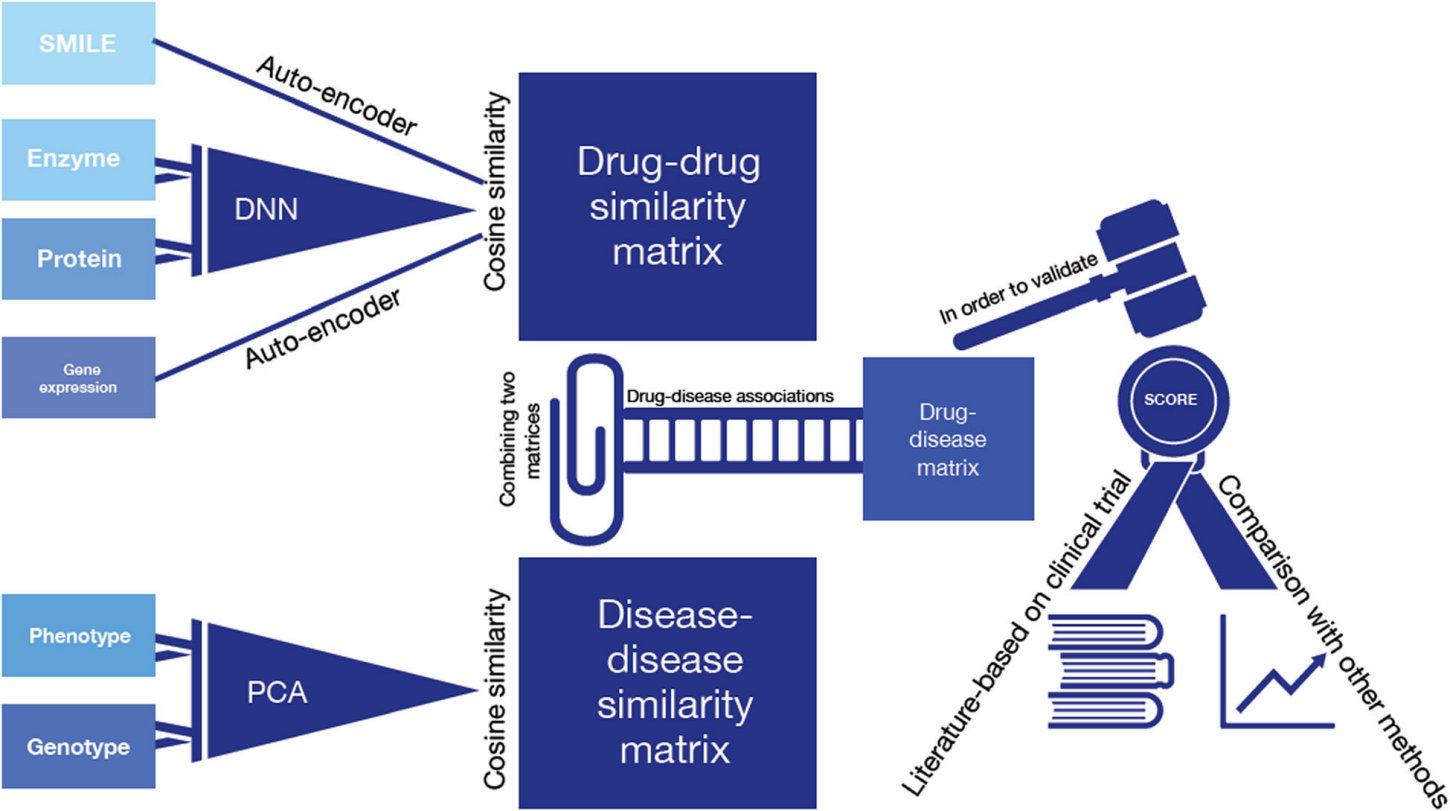
The pipeline of our steps in our approach

In this section, we follow the five steps (see Fig. 1) to find new indications for existing drugs (drug repositioning):

1. Representing four drug features using deep neural network.
2. Transforming two disease features represented by one-hot-encoder using PCA.
3. Using drug features to construct the drug-drug similarity matrices.
4. Using disease features to construct the disease-disease similarity matrices.
5. Using drug-drug similarity and disease-disease similarity to construct drug-disease association matrices.

### Representing four drug features using deep neural network

In this subsection, we extract four drug features, chemical structures, protein sequences of drug targets, drug-related enzyme sequences and gene expression profiles. Also, the appropriate representation of features, derived by deep neural networks, is introduced.

### Chemical structures

Numerous studies have attempted to explain the importance of chemical structures [8]. For instance, SMILES simplifies the chemical structure and encodes molecular graphs compactly as a human-readable string and describes molecules with an alphabet of characters as a formal grammar [41]. We download the SMILES strings from the DrugBank [42] and PubChem [43] database during the 2017–2018 academic year.

We use the variational auto-encoder (VAE) [38] to convert the discrete representation of molecules (SMILES string) into a continuous 192-dimensional vector. The SMILES string of drug *i* is pre-processed by the following steps to make appropriate inputs for VAE model:

- A subset of 35 different characters is used for SMILES-based text encoding.
- The strings are encoded up to a maximum length of 120 characters. Some spaces are added to shorter strings in order for all strings to be the same length.

Finally, the pre-processed SMILES string of drug *i* is given as an input to VAE model and vector ${\overrightarrow{s}}_i$ is generated as an appropriate representation named SMILES vector. The “Keras” [44] and “Theano” packages [45] are utilized to apply this neural net.

### Protein sequences of drug target

Each drug addresses one or multiple drug targets, which is a molecule associated with a particular disease process, to produce a desired therapeutic effect [46]. Drug targets are mostly proteins with active sites which can be ducked to the drugs. Each drug has one or multiple target proteins, and each protein can be the potential target of multiple drugs.

We retrieve drug target protein sequences from DrugBank during the 2017–2018 academic year [42]. We download the drug target section that includes proteins and genes. In this database, there is a list of drugs for each protein. Thus, we list the sequences of the target proteins for each drug.

We apply a deep neural network model named ProtVec [39] to convert the protein sequence into three continuous 100-dimensional vectors. In other words, each protein sequence is represented as three sequences of 3-gram. In n-gram modelling of protein informatics, usually, an overlapping window of 3 to 6 residues is used. ProtVec [39], instead of taking overlapping windows, generates three vectors of shifted non-overlapping words. Each 3-gram is presented as a vector of size 100.

For each drug *i*, we perform the following steps to generate a set of 300-dimensional vectors called *ℙ*_*i*_ to represent the sequences of target proteins:

- The sequences of target proteins are listed as a set named Φ_*i*_ where |Φ_*i*_| shows the number of targeted proteins by the drug *i*.
- Each protein sequence *σ* ∈ Φ_*i*_ is given as an input to ProtVec. Three 100-dimensional vectors named $\overrightarrow{{v_1}^{\sigma }}$, $\overrightarrow{{v_2}^{\sigma }}$ and $\overrightarrow{{v_3}^{\sigma }}$ are generated as outputs.
- For protein sequence *σ*, the concatenation of these 3 vectors is computed as $\overrightarrow{\ {v}^{\sigma }}=\overrightarrow{{v_1}^{\sigma }}.\overrightarrow{{v_2}^{\sigma }}.\overrightarrow{{v_3}^{\sigma }}$.
- Drug *i* is represented by the associated proteins of set Φ_*i*_ as $\kern0.50em {\mathbb{P}}_i=\left\{\overrightarrow{v^{\sigma }}|\sigma \in {\Phi}_i\right\}$.

### Drug-related enzyme sequences

Drug-related enzyme sequences include all the enzymes involved in the activation and metabolism of a drug. We extract these sequence from DrugBank during the 2017–2018 academic year [42]. For each drug *i*, we execute the same process explained in section "Protein sequences of drug target" for enzyme sequences to generate a continuous 300-dimensional vectors based on drug-related enzymes called ${\mathbbm{E}}_i$.

### Gene expression profiles

We obtain raw data of gene expression profiles (GEPs) of CMAP dataset [12], and normalize them using R/Bioconductor “affy” package. These samples contain GEPs of five cell lines, either untreated or treated with any of 1309 different drugs. Differential gene expression profile (dGEP) of each cell line in presence vs. absence of a drug is computed by subtracting log2-scaled GEPs after merging biological replicated samples via mean function. A subset of 729 drugs are annotated and approved in Drug Bank [42] and PubChem [43] databases.

We use a specific architecture of stacked auto-encoders in a number of previous researches [47, 48]. It was shown, this architecture can retrieve important biological features of the data, such as gene co-expression patterns, pathways and biological processes [47], and exploit them to reduce the dimensionality of GEPs into a footprint sized vector called cell identity code (CIC) that contains important features of the data [48]. Importantly, CICs are resistant to noise and missing data [48] and can prevent overfitting by reducing the number of parameters of a deep neural network, when they are used as the input rather than the original GEPs.

**Fig. 2:**
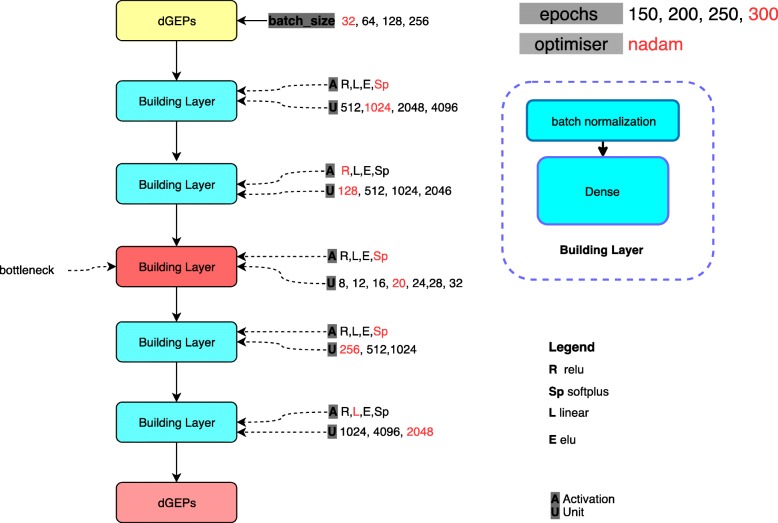
The hyper-parameters. The best values of parameters (batch size, epochs, unit, activation and optimizer) are determined by red colour. The yellow box shows the input of network (differential gene expression profiles). The blue box represents each layer of the network. The red box (bottleneck), illustrates the best representation of dGEPs. The pink box identifies the predicted dGEPs from the bottleneck representation

For these reasons, we design a stacked auto-encoder of five layers, after observing that increasing the number of layers did not impact on decreasing the loss function. For each layer, different options for the number of neurons and the activation functions are listed, as potential values for hyper-parameters. Then we use a Bayesian approach for hyper-parameter optimization using “hyperopt” package [49]. Different options for activation function are rectified linear unit (ReLU), Linear, SoftPlus, and ELU. The optimal value for batch size is also selected through hyper-parameter optimization. Different options for each hyper-parameter are specified in Fig. 2. The learning rate is 0.001. We use mean square error (MSE) as the regression loss-function. “nadam” algorithm is used for both hyper-parameter optimization and final training.

We partition the data into training (60%), validation (15%) and test (25%) datasets. The stacked auto-encoder is trained and the appropriate weights and bias values are found. The validation dataset is used for hyper-parameter optimization. The test dataset is utilized for final evaluation of the model.

We perform 100 iterations of hyper-parameter optimization. The final hyper-parameters that were selected by the optimization process are highlighted in Fig. 2. After performing 300 epochs iteration, the optimal candidate network has the mean-squared error of 0.076.

Subsequently, the output of the bottleneck layer for available differential expression profiles has been extracted with the mean-squared error of about 0.0047 as loss and mean absolute error of around 0.0495. The output of this auto-encoder is a 20-dimensional vector representing dGEP ($\overrightarrow{g_i}$).

### Transforming two disease features represented by one-hot-encoder using PCA

In order to find disease-disease similarity, we employ two sets of measures, namely the phenotypes (characteristics of a disease) and genotypes (genes involved in a disease). We download 10,881 human diseases with 8662 phenotypes and 7217 human diseases with 10,764 genotypes from Monarch [50]. In their intersection, there are 5955 diseases with both phenotypes and genotypes. For disease *i*, two one-hot-encoders, namely 8662-dimensional and 10,764-dimensional vectors, are constructed for phenotype and genotype, respectively.

For disease *i*, a phenotype one-hot-encoder is a zero vector with length 10,881. If a phenotype belongs to the disease, then the corresponding component of the vector is substituted 1. Also, we make genotype one-hot-encoder similar to phenotype one-hot-encoder.

These two one-hot-encoders are too sparse, specifically the one regarding genotype. To overcome this issue, we generate two vectors called $\overrightarrow{{\mathrm{a}}_{\mathrm{i}}}$ and $\overrightarrow{{\mathrm{d}}_{\mathrm{i}}}$ for phenotype and genotype using PCA, respectively. By test and trial, we find out appropriate numbers of components for PCA that identify the length of vectors $\overrightarrow{{\mathrm{a}}_{\mathrm{i}}}$ and $\overrightarrow{{\mathrm{d}}_{\mathrm{i}}}$ with 30 and 20, respectively.

### Using drug features to construct the drug-drug similarity matrices

In this subsection, we generate a similarity matrix for each drug feature. We assume that there are *n* drugs. For each drug *i*, there are two vectors called $\overrightarrow{{\mathrm{s}}_{\mathrm{i}}\ }$, $\overrightarrow{{\mathrm{g}}_{\mathrm{i}}}$ and two sets named *ℙ*_i_, ${\mathbbm{E}}_{\mathrm{i}}$ to show the representation of chemical structures (*s*), gene expression profiles (*g*), protein sequences of drug target (*p*) and drug-related enzyme sequences (*e*), respectively.

We make a similarity matrix for each feature *x* ∈ {*s*, *g* } named ${M}_{n\times n}^x$, the value of *n* shows the number of drugs, as follows:

$$ {M}^x\left[i,j\right]= sim\left(\overrightarrow{x_i},\overrightarrow{x_j}\right), $$

where the feature *x* is available for drug *i* in the database. The similarity between drugs *i* and *j* based on feature *x* is computed by *sim* function using Cosine measures which is more compatible with our data [51]. In order to compute *sim* function, we use the “proxy” package in R [52].

In addition, we make a similarity matrix ${\mathrm{M}}_{\mathrm{n}\times \mathrm{n}}^{\mathrm{p}}$ for protein sequences of drug targets as follows:

1. *ℙ*_i_ and *ℙ*_j_ are made as it was mentioned in section "Protein sequences of drug target".

If |*ℙ*_i_| ≤  ∣ *ℙ*_j_ ∣ ,

$$ \forall \overrightarrow{\uprho_{\mathrm{i}}}\in {\mathbb{P}}_{\mathrm{i}},\kern1em {\mathrm{R}}_{\overrightarrow{\uprho_{\mathrm{i}}}}=\underset{\ \overrightarrow{\uprho_{\mathrm{j}}}\in {\mathbb{P}}_{\mathrm{j}}}{\max}\mathrm{sim}\left(\ \overrightarrow{\uprho_{\mathrm{i}}},\overrightarrow{\uprho_{\mathrm{j}}}\right),{\mathrm{M}}^{\mathrm{p}}\left[\mathrm{i},\mathrm{j}\right]={\sum}_{\overrightarrow{\uprho_{\mathrm{i}}}\in {\mathbb{P}}_{\mathrm{i}}}{\mathrm{R}}_{\overrightarrow{\uprho_{\mathrm{i}}}} $$ .

If |*ℙ*_i_| >  ∣ *ℙ*_j_ ∣ ,

$$ \forall \overrightarrow{\uprho_{\mathrm{j}}}\in {\mathbb{P}}_{\mathrm{j}},\kern1em {\mathrm{R}}_{\overrightarrow{\uprho_{\mathrm{j}}}}=\underset{\ \overrightarrow{\uprho_{\mathrm{i}}}\in {\mathbb{P}}_{\mathrm{i}}}{\max}\mathrm{sim}\left(\ \overrightarrow{\uprho_{\mathrm{i}}},\overrightarrow{\uprho_{\mathrm{j}}}\right),{\mathrm{M}}^{\mathrm{p}}\left[\mathrm{i},\mathrm{j}\right]={\sum}_{\overrightarrow{\uprho_{\mathrm{j}}}\in {\mathbb{P}}_{\mathrm{j}}}{\mathrm{R}}_{\overrightarrow{\uprho_{\mathrm{j}}}} $$ .

According to the set of drug-related enzyme sequences, the similarity matrix between drugs i and j, M^e^[i, j], is constructed like the protein sequences of drug targets.

In the following, drug-drug similarity intersection (DDSI) matrix called ${I}_{n\times n}^E$ is constructed on the subset *E* ⊆ {*s*, *p*, *e*, *g*}. The number of drugs (*n*) shows that all features of the set *E* is available in the database:

$$ {I}^E\left[i,j\right]=\Big\{{\displaystyle \begin{array}{c}\left(\sum \limits_{x\in E}{M}^x\left[i,j\right]-\min \right)/\left(\max -\min \right),\kern0.5em i\ne j\\ {}1\kern14.5em ,\kern0.5em else\end{array}}\operatorname{} $$

where

$$ \mathit{\min}=\underset{1\le i\ne j\le n}{\mathit{\min}}\sum \limits_{x\in E}{M}^x\left[i,j\right]-0.01, $$

and

$$ \mathit{\max}=\underset{1\le i\ne j\le n}{\mathit{\max}}{\sum}_{x\in E}{M}^x\left[i,j\right]+0.01. $$

### Using disease features to construct the disease-disease similarity matrices

We assume that there are *m* diseases. For each disease *i*, there are two vectors called $\overrightarrow{a_i}$ and $\overrightarrow{d_i}$ to show the representation of phenotype (*a*) and genotype (*d*) respectively. We display the length of these vectors below:

$$ \left|\overrightarrow{a_i}\right|=30,\left|\overrightarrow{d_i}\ \right|=20. $$

We make a similarity matrix for each feature *x* ∈ {*a*, *d* } named ${M}_{m\times m}^x$ as follows:

$$ {M}^x\left[i,j\right]= sim\left(\overrightarrow{x_i},\overrightarrow{x_j}\right), $$

where *sim* function shows the similarity between diseases *i* and *j* based on feature *x* using Cosine measure [51]. In order to compute the *sim* function, we use the “proxy” package in R [52]. Finally, the disease-disease similarity (DiDiS) matrix called *D*_*m* × *m*_ is constructed as follows:

$$ D\left[i,j\right]=\Big\{{\displaystyle \begin{array}{c}\left(\sum \limits_{x\in \left\{a,d\right\}}{M}^x\left[i,j\right]-\min \right)/\left(\max -\min \right),\kern0.5em i\ne j\\ {}1\kern15em ,\kern0.5em else\end{array}}\operatorname{} $$

where

$$ \mathit{\min}=\underset{1\le i\ne j\le n}{\mathit{\min}}\sum \limits_{x\in \left\{a,d\ \right\}}{M}^x\left[i,j\right]-0.01, $$

and

$$ \mathit{\max}=\underset{1\le i\ne j\le n}{\mathit{\max}}{\sum}_{x\in \left\{a,d\ \right\}}{M}^x\left[i,j\right]+0.01. $$

### Using drug-drug similarity and disease-disease similarity to construct drug-disease association matrices

In this subsection, we define the drug-disease association (DDA) matrix ${A}_{n\times m}^E$ where E is a subset of drug features. To do this, we apply DDSI matrix ${I}_{n\times n}^E$ and DiDiS matrix *D*_*m* × *m*_ to generate ${A}_{n\times m}^E$ as follows [29]:

1 $$ {A}^E\left[i,j\right]={\mathit{\operatorname{Max}}}_{\begin{array}{c}\left({i}^{\prime },{j}^{\prime}\right)\in \mathcal{A}\\ {}i\ne {i}^{\prime },\kern0.5em j\ne {j}^{\prime}\end{array}}\sqrt{I^E\left[i,i^{\prime}\right]\times D\left[j,j^{\prime}\right]}\kern0.5em $$

where each pair (*i*^′^, *j*′) is selected from the previously known drug-disease associations set $\mathcal{A}$.

To make the drug-disease association matrices (*A*^*E*^), we assemble the known drug-disease associations (set $\mathcal{A}$) from repoDB [53] and Zhang et al. [30] Datasets.

## Results

In this section, we find the best subset of drug features for drug repositioning. Then our method is compared with some computational methods.

Table 1 illustrates the details of the data set where the first and second columns show each subset of drug features and the number of drugs which these features are available in the database, respectively. The third column indicates the number of drug-disease associations where the features are available in the database and the fourth one identifies the number of unknown drug-disease associations corresponding to each combination of drug features.

**Table 1:** The first and second columns show each subset of drug features and the number of drugs which these features are available in the database, respectively. The third column indicates the number of drug-disease associations where the features are available in the database and the fourth one identifies the number of unknown drug-disease associations

Subset *E*	No. of Drugs	No. of drug associations ($\mathcal{A}$)	No. of unknown drug-disease association	Avg. of AUC	AUC
{s}	4240	13,916	25,235,284	0.942	0.944
{g}	729	6175	4,335,020	0.894	0.888
{e}	671	10,950	3,984,855	0.927	0.926
{p}	6233	16,846	37,100,669	0.942	0.943
{g,s}	729	6175	4,335,020	0.936	0.933
{e,s}	471	8398	2,796,407	0.870	0.871
{s,p}	3226	13,159	19,197,671	0.941	0.941
{e,g}	155	4065	918,960	0.856	0.844
{g,p}	337	5928	2,000,907	0.857	0.864
{e,p}	600	10,305	3,562,695	0.909	0.906
{e,g,s}	155	4065	918,960	0.849	0.848
{g,p,s}	337	5928	2,000,907	0.876	0.877
{g,p,e}	146	3944	865,486	0.834	0.844
{e,p,s}	440	8162	2,612,038	0.868	0.870
{s,e,g,p}	146	3944	865,486	0.840	0.846

### Drug features assessment

A cross-validation scheme called leave-one-out is used to find the best subset of drug features for drug repositioning. We predict the association of drug *i* and disease *j* based on known associations (see eq. 1). In other words, we hide the known association of drug *i* and disease *j*, then use the other known associations to score this pair.

We compute the area under the curve (AUC) for the following test data to evaluate our method. The positive and negative sets of the test data are defined based on 10% of predicted known and unknown drug-disease association pairs obtained from the matrix ${A}_{n\times m}^E$, respectively. This process is repeated for twenty times to make the test set. The average AUC is shown in the fifth column of Table 1.

To show that the size of the negative set has a negligible effect on the AUC score, we make a test set from all predicted known and unknown drug-disease association pairs obtained from the matrix ${A}_{n\times m}^E$. The number of positive and negative data of these test sets can be seen in the third and fourth columns of Table 1. The AUC value is in the sixth column, and close to the fifth one. The results show that all drug features are profitable for drug indication prediction (see Table 1). The table shows that  {*s*}, {*p*}, {*e*}, {*g*, *s*}, {*s*, *p*} and {*e*, *p*} subsets are more informative than the other subsets of drug features; however, we cannot ignore the positive impact of the number of associations related to each subset.

For further discussion, we assess each subset of drug features to find out which drugs are effective for a specific disease and which diseases are treatable by a particular drug.

We extract 585 diseases which are in the known drug-disease associations (set $\mathcal{A}$) related to 146 drugs, including all features. For each subset of drug features, the AUC value of each disease is calculated, and then the average of AUCs is shown in the second column in Table 2. The second column of Table 2 shows {s}, {g, s}, and {s, p} subsets are appropriate to find which drugs are effective for a specific disease. Chemical structure ( SMILES) feature is common among these subsets. This is why so many pharmaceutical companies [8] have been using this feature to find new indications.

**Table 2:** The second and third columns show the average and standard deviation of AUCs on 585 diseases and 137 drugs for each subset of drug features, respectively

Subset *E*	*Avg* ∓ *STDV* of AUC on 585 disease	*Avg* ∓ *STDV*of AUC on 137 drugs
{s}	0.909 ∓ 0.08	0.802 ∓ 0.14
{g}	0.724 ∓ 0.18	0.837 ∓ 0.10
{e}	0.495 ∓ 0.19	0.921 ∓ 0.09
{p}	0.620 ∓ 0.22	0.939 ∓ 0.05
{g,s}	0.911 ∓ 0.08	0.790 ∓ 0.15
{e,s}	0.821 ∓ 0.11	0.795 ∓ 0.15
{s,p}	0.896 ∓ 0.09	0.807 ∓ 0.14
{e,g}	0.644 ∓ 0.20	0.839 ∓ 0.11
{g,p}	0.713 ∓ 0.19	0.836 ∓ 0.11
{e,p}	0.570 ∓ 0.20	0.920 ∓ 0.06
{e,g,s}	0.797 ∓ 0.14	0.792 ∓ 0.15
{g,p,s}	0.833 ∓ 0.12	0.798 ∓ 0.14
{g,p,e}	0.687 ∓ 0.19	0.832 ∓ 0.12
{e,p,s}	0.822 ∓ 0.11	0.797 ∓ 0.14
{s,e,g,p}	0.798 ∓ 0.14	0.792 ∓ 0.15

The intersection of known drug-disease association (set $\mathcal{A}$) with the list of drugs, including all features is 137 drugs. AUC value of each drug is calculated for each subset of drug features and then the average of AUCs is shown in the third column of Table 2. The third column shows {e}, {p} and {e, p} subsets are proper to identify which diseases are treatable with a specific drug.

Drug-related enzyme sequences (*e*) are informative, including all the enzymes involved in the activation and metabolism of a drug. Metabolism of drugs in the body is a complex process where drugs are structurally modified to different molecules (metabolites) by various metabolizing enzymes. Studies on drug metabolism are key processes to safety profiles of drug candidates in drug discovery and development [54]. Meanwhile, protein sequences of drug target (*p*) are known as an essential feature for drug repositioning due to similar binding sites may bind to similar drugs as an assumption [55].

### Comparison with some computational methods

We compare our pipeline with three different state-of-the-art methods using five-fold cross-validation [5, 30, 31]. To further analysis, we extract some specific diseases to comparison with two network-based methods [21, 40].

### Comparison with two network-based approaches on some specific diseases

We compared our pipeline with two network-based approaches [21, 40]. We extract 21 common diseases of Yang & Agarwa1 [40] and Lee [21] to evaluate our pipeline. We perform our pipeline based on appropriate subsets of drug features ({s}, {g, s}, and {s, p}) to find which drugs are effective for a specific disease (see section "Drug features assessment"). The third to sixth columns of Table 3 show the AUC values of Yang & Agarwa1 and Lee approaches. The last three columns represent the AUC values of each disease obtained by our pipeline. The average AUCs of Yang & Agarwa1 network, Random forest, N-Net and three different versions of our pipeline are 0.66, 0.76, 0.68, 0.89 and 0.87, respectively in Table 3.

**Table 3:** Comparison three different versions of our pipeline with Yang & Agarwal [40] and Lee [21] on 21 diseases

MONDO	Disease name	Yang & Agarwa1	Lee (Random forest)	Lee (N-Net)	Ours {*s*}	Ours {*g*, *s*}	Ours {*s*, *p*}
0000190	ventricular fibrillation	0.74	0.85	0.78	0.81	0.82	0.79
0001627	dementia	0.62	0.89	0.79	0.83	0.89	0.81
0002049	thrombocytopenia	0.50	0.67	0.72	0.95	0.95	0.94
0002243	hemorrhagic disease	0.59	0.69	0.67	0.97	1.00	0.96
0003620	peripheral nervous system disease	0.91	0.64	0.69	0.92	0.93	0.91
0004975	alzheimer disease	0.68	0.62	0.61	0.86	0.89	0.84
0004976	amyotrophic lateral sclerosis	0.58	0.73	0.59	0.96	0.98	0.95
0004979	asthma	0.53	0.73	0.68	0.73	0.85	0.69
0004981	atrial fibrillation	0.50	0.80	0.79	0.87	0.92	0.85
0004985	bipolar disorder	0.69	0.84	0.82	0.87	0.90	0.86
0005015	diabetes mellitus	0.66	0.79	0.71	0.92	0.89	0.91
0005027	epilepsy	0.62	0.75	0.70	0.81	0.87	0.79
0005041	glaucoma	0.60	0.85	0.58	0.90	0.93	0.89
0005059	leukemia	0.69	0.79	0.55	0.97	0.97	0.97
0005062	lymphoma	0.72	0.85	0.55	0.97	0.94	0.97
0005068	myocardial infarction	0.64	0.70	0.68	0.92	0.91	0.91
0005180	parkinson disease	0.70	0.74	0.69	0.81	0.86	0.78
0005275	lung disease	0.70	0.78	0.68	0.94	0.90	0.93
0005578	arthritis	0.67	0.73	0.52	0.91	0.92	0.90
0008114	obsessive-compulsive disorder	0.95	0.79	0.76	0.97	0.95	0.97
0011122	obesity	0.64	0.72	0.66	0.67	0.44	0.71

### Comparison with some state-of-the-art methods

A five-fold cross-validation scheme is used to evaluate the accuracy of our pipeline based on the chemical structure of a drug. The AUC value of our model is 0.935 and it is comparable with PREDICT (AUC = 0.902) [5], SCMFDD (AUC = 0.920) [30] and DisDrugPred (AUC = 0.922) [31].

The prediction part of our method acts like PREDICT. Here, we describe the differences between PREDICT and our pipeline. First, we use deep neural networks to reduce the dimensionality of data [56] for extracting drug features and PCA for disease features to find an efficient representation. Second, we collect broader drug-disease associations set than PREDICT. Finally, this pipeline is scalable, and we observe the semantic relations between drugs and diseases, even using only one of the drug features.

## Discussion

In this section, we investigate clinical trial studies for several predicted drug-disease pairs showing high probabilities among our prediction [57]. In other words, to evaluate our efficiency and performance, we assess our results to discover the potential drug- disease associations with some clinical trial studies that have been published before by database records [57]. The top repositioning candidates from our pipeline analysis are listed in Table 4.

**Table 4:** New drug-disease associations score obtained by our pipeline

Drug name	Disease name	MONDO	Drug-Bank ID	Score	Reference
Asthma	Budesonide	0004979	DB01222	0.962	https://ClinicalTrials.gov/show/NCT03034005
Addison Disease	Dexamethasone	0009410	DB01234	0.938	https://ClinicalTrials.gov/show/NCT03210545
Lupus Nephritis	Mycophenolate Mofetil	0005556	DB00688	0.936	https://ClinicalTrials.gov/show/NCT03920059
Cancer	Dexamethasone	0004992	DB01234	0.931	https://ClinicalTrials.gov/show/NCT02815319
Hypothyroidism	Levothyroxine	0005420	DB00451	0.913	https://ClinicalTrials.gov/show/NCT02577367
Paroxysmal Nocturnal Hemoglobinuria	sirolimus	0018641	DB00877	0.876	https://ClinicalTrials.gov/show/NCT03866681
Multiple Sclerosis	Fingolimod	0005301	DB08868	0.843	https://ClinicalTrials.gov/show/NCT02232061
Peripheral Arterial Disease	Ramipril	0005386	DB00178	0.843	https://ClinicalTrials.gov/show/NCT02842424
Chronic Hepatitis b	Tenofovir Alafenamide	0005366	DB09299	0.827	https://ClinicalTrials.gov/show/NCT03753074
Kidney Disease	Dexmedetomidine	0005240	DB00633	0.825	https://ClinicalTrials.gov/show/NCT02707809
Multiple Sclerosis	Cladribine	0005301	DB00242	0.821	https://ClinicalTrials.gov/show/NCT03961204
Asthma	N-acetylcysteine	0004979	DB06151	0.809	https://ClinicalTrials.gov/show/NCT03581084
Peutz-Jeghers Syndrome	Rapamycin	0008280	DB00877	0.807	https://ClinicalTrials.gov/show/NCT03781050
Malaria	primaquine	0005136	DB01087	0.799	https://ClinicalTrials.gov/show/NCT03916003
Alopecia Areata	Tofacitinib	0005340	DB08895	0.777	https://ClinicalTrials.gov/show/NCT03800979
Multiple Sclerosis	Fampridine	0005301	DB06637	0.766	https://ClinicalTrials.gov/show/NCT02849782

## Conclusions

In this article, we presented a pipeline for drug repositioning based on a non-linear computational approach. We consider four different drug features named the chemical structure of drugs, protein sequences of drug target, drug-related enzyme sequences, and gene expression profiles. In addition, two features, called phenotype and genotype, are considered for diseases. Efficient representation of data enables integrative analysis and reduces the dimension and heterogeneity of drug and disease features. To find appropriate representation, we use deep learning model to generate some continuous vectors for drug and disease features. Based on these vectors, we make a drug-disease similarity matrix to predict new drug indications. The result showed that our method predicts new drug-disease associations systematically in a more cost-effective way and shorter timeline.

This pipeline can see the semantic relations between drugs and diseases using only one drug feature, which means every single one of drug features is informative. This pipeline is scalable and acts as a viable strategy for merely identifying and developing new therapeutic uses for existing or abandoned pharmacotherapies.

## Data Availability

All DDA matrices are available in http://bioinformatics.aut.ac.ir/drug-disc/.

## Abbreviations

AUC: The area under the receiver operating characteristic curve
CIC: Cell identity code
CMAP: The connectivity map
DDA: Drug-disease association matrix
DDSI: Drug-drug similarity intersection matrix
dGEP: Differential gene expression profile
DiDiS: Disease-disease similarity matrix
ELU: Exponential linear unit
GEPs: Gene expression profiles
MSE: Mean square error
PCA: Principal component analysis
ReLU: Rectified linear unit
SDF: Subset of drug features
SMILES: Simplified molecular-input line-entry system
VAE: Variational auto-encoder
